# A Tolman-like Compact Model with Conformal Geometry

**DOI:** 10.3390/e23111406

**Published:** 2021-10-26

**Authors:** Didier Kileba Matondo, Sunil D. Maharaj

**Affiliations:** 1 Astrophysics Research Centre, School of Mathematics, Statistics and Computer Science, University of KwaZulu-Natal, Private Bag X54001, Durban 4000, South Africa; didier.kileba@unikin.ac.cd; 2 Department of Physics, University of Kinshasa, Kinshasa, Democratic Republic of the Congo

**Keywords:** tolman geometry, conformal symmetry, compact objects

## Abstract

In this investigation, we study a model of a charged anisotropic compact star by assuming a relationship between the metric functions arising from a conformal symmetry. This mechanism leads to a first-order differential equation containing pressure anisotropy and the electric field. Particular forms of the electric field intensity, combined with the Tolman VII metric, are used to solve the Einstein–Maxwell field equations. New classes of exact solutions generated are expressed in terms of elementary functions. For specific parameter values based on the physical requirements, it is shown that the model satisfies the causality, stability and energy conditions. Numerical values generated for masses, radii, central densities, surface redshifts and compactness factors are consistent with compact objects such as PSR J1614-2230 and SMC X-1.

## 1. Introduction

An interesting suggestion to gain a deeper understanding of self-gravitating systems is to apply the idea of complexity in theories of gravity. An approach that was suggested in this direction is due to Herrera [1] characterized by a minimal complexity factor. This approach is also applicable to the study of compact objects and neutron stars in general relativity. Several studies have been made involving complexity in models arising in general relativity [2,3,4,5,6,7,8,9,10]. Note that the idea of complexity may be applied to extended theories of gravity [11,12]. Recently Jasim et al. [13] showed that a strange star in Einstein–Gauss–Bonnet gravity can be developed which is consistent with complexity. A general geometric concept that may also be used to study relativistic self-gravitating fluids is a symmetry on the manifold. The conformal Killing vector symmetry has been applied to particular stellar models in the past. Therefore, in this paper, we consider the existence of a conformal Killing vector for a static spacetime with a charged matter distribution. Our results show that a conformal symmetry is indeed useful in generating a relativistic stellar model. This may assist in producing a general relationship between a conformal symmetry, and possibly a general symmetry, and complexity of a gravitating model.

In the search for interior exact solutions to the Einstein field equations in static spherically symmetric spacetimes, several results have been generated since the first interior solution was found by Schwarzschild. Tolman [14] proposed an innovative method of treatment for the Einstein field equations which resulted in a variety of new solutions, known as the Tolman I, II, III, IV, V, VI, VII and VIII cases. Many of these solutions have been studied in the past to obtain stellar models by imposing an equation of state in the field equations. Some of these models are physically significant. Special interest has been paid to the Tolman VII metric function because the most desirable features of this solution result from regularity inside the star which is free of singularity at the center. Delgaty and Lake [15] discussed families of exact solutions to the Einstein field equations and pointed out the most important criteria to be satisfied for a realistic stellar model. Hensh and Stuchlik [16] obtained a new anisotropic model with the help of the Tolman VII metric function and the minimal geometric deformation approach, satisfying all physical requirements for a realistic relativistic star. The Tolman VII metric function has been used in a number of works, including the case when the interior of static spherically symmetric spacetime is anisotropic or admits the presence of charge. Physically reasonable models with Tolman VII geometry admitting linear and quadratic equations of state have been investigated by Bhar et al. [17,18]. Singh et al. [19] discussed the effects of exotic matter in the compact object by using the Tolman VII solution with a generalized nonlinear equation of state. The presence of anisotropy and charge in the stellar interior influences the physical properties, stability and equilibrium of the model. Recently solutions of the Einstein–Maxwell field equations have been found with Tolman VII spacetime for charged anisotropic fluid distributions by Singh et al. [20], Malaver [21,22] and Kiess [23]. Note that Raghoonundun and Hobill [24] revisited the Tolman VII solution by providing a set of parameters which provides a class of equations of state in closed form.

Historically the search for exact solutions of the Einstein field equations involved many mathematical techniques, imposing an equation of state, specifying the gravitational potentials and imposing a spacetime symmetry. Recently, it has been shown that the presence of a conformal symmetry can also lead to a stellar model. The conformal symmetry requires the existence of a conformal Killing vector on the spacetime manifold. It is interesting to note that the resulting conformal factor is purely geometric and independent of the form of the energy momentum tensor. Remarkably, the solutions obtained via this geometric method describe compact objects such as strange stars to a very good approximation. In particular, the conformal symmetry vector places restrictions on the gravitational potentials which can be used to generate exact solutions of the Einstein field equations. It is an alternative approach where the gravitational metrics are used rather than restricting the energy momentum. Detailed studies involving conformal motions in static spherically symmetric spacetimes have been performed recently by Tupper et al. [25] and Maartens et al. [26,27]. Manjonjo et al. [28,29] used the Weyl tensor for conformally flat and non-conformally flat spacetimes to establish a relationship between the static metric potentials. An extensive investigation of exact models for compact objects has been performed by Mafa Takisa et al. [30] and Kileba Matondo et al. [31,32] with the help of this conformal relationship. Some other authors such as Maurya et al. [33], Esculpi and Aloma [34], Singh et al. [35], Rahaman et al. [36], Shee et al. [37] and Deb et al. [38] emphasized the importance of conformal invariance in compact stars.

The Einstein–Maxwell equations for a spherically symmetric spacetime are nonlinear, and they cannot be solved in general without simplifying assumptions. In many treatments a specific choice for the gravitational potentials or matter variables are made; the field equations are then solved. This approach is arbitrary. An alternative approach is to impose a spacetime symmetry requirement on the manifold. An interesting symmetry that exists is generated by a conformal Killing vector which maps null geodesics to null geodesics and produces conserved quantities for massless particles. The existence of a spacetime symmetry provides a geometric characterization of the model. In addition, the conformal Killing vector produces models which are physically realistic [30,31,32,33,34,35,36,37,38]. The existence of a conformal Killing vector in spherically symmetric spacetimes gives a relationship between the gravitational potentials [28,29].This provides a systematic approach of generating new exact solutions to the Einstein–Maxwell field equations. Stellar models generated in this approach, characterized by a spacetime symmetry, are physically acceptable and produce values for masses and radii corresponding to observed relativistic stars.

In this investigation, we aim to generate a new class of charged anisotropic solutions for compact stars invariant under conformal symmetry via the generalized condition of anisotropic pressure established in Manjonjo et al. [29]. Most of the solutions for compact stars found with conformal symmetry are not regular at the center. The Tolman VII metric function can be used to integrate the conformal Killing condition of anisotropic pressure with the help of particular forms of the electric field functions to generate new classes of exact solutions which are free from singularities. This work is structured as follows. In Section 2, we provide the Einstein–Maxwell field equations for charged anisotropic matter distribution. New classes of exact solutions to the Einstein–Maxwell system are generated with the help of the condition of anisotropic pressure in Section 3. Physical quantities arising from one of the classes of solutions selected are presented in Section 4. Geometrical and matter variables are plotted for both PSR J1614-2230 and SMC X-1 in Section 5 and detailed physical features are also studied. In Section 6 the work is concluded.

## 2. Field Equations

The line element describing the interior of a static relativistic star can be written as

(1) $$ds^{2}=-e^{2\nu (r)}dt^{2}+e^{2\lambda (r)}dr^{2}+r^{2}\left(d\theta^{2}+\sin^{2}\theta d\phi^{2}\right),$$

where λ(r) and ν(r) represent gravitational potential functions. The matter distribution in the stellar interior is assumed to be charged and anisotropic with the energy momentum tensor

(2) $$\begin{array}{c} T_{ab}=\mathrm{diag}\left(-\rho -\frac{1}{2}E^{2},p_{r}-\frac{1}{2}E^{2},p_{t}+\frac{1}{2}E^{2},p_{t}+\frac{1}{2}E^{2}\right), \end{array}$$

where the quantities ρ, $p_{r}$, $p_{t}$ and *E* denote the energy density, radial pressure, tangential pressure and electric field intensity, respectively. With the help of (1) and (2), we get the Einstein–Maxwell system of equations

(3a)1r2r(1−e−2λ)′=ρ+12E2,(3b)−1r2(1−e−2λ)+2ν′re−2λ=pr−12E2,(3c)e−2λν″+ν′2+ν′r−ν′λ′−λ′r=pt+12E2,(3d)σ2=1r2(r2E)′e−λ,

in terms of the radial coordinate *r*, and primes denote the derivative with respect to *r*. Here, we assume units in which 8πG=c=1.

We introduce the following transformation:

(4) $$\begin{array}{c} x=Cr^{2},Z\left(x\right)=e^{-2\lambda (r)},A^{2}y^{2}\left(x\right)=e^{2\nu (r)}, \end{array}$$

where A and *C* are constants. Then, the metric (1) becomes

(5) $$\begin{array}{ccc} ds^{2} & = & -A^{2}y^{2}dt^{2}+\frac{1}{4CxZ}dx^{2}+\frac{x}{C}\left(d\theta^{2}+\sin^{2}\theta d\phi^{2}\right). \end{array}$$

The above expressions are due to Durgapal and Bannerji [39] and can be used to obtain an equivalent form of the system (3) with new variables. We now rewrite the Einstein–Maxwell field Equation (3) as

(6a)ρC=−2Z˙+1−Zx−E22C,(6b)prC=4Zy˙y+Z−1x+E22C,(6c)ptC=4xZy¨y+(4Z+2xZ˙)y˙y+Z˙−E22C,(6d)σ2C=4Zx(xE˙+E)2,

where dots now denote differentiation with respect to *x*. The mass contained within a radius *x* of the relativistic anisotropic compact star is

(7) $$M\left(x\right)=\frac{1}{C^{3/4}}\int_{0}^{x}\sqrt{\omega }\left(\rho_{unch}\left(\omega \right)+E^{2}\right)d\omega ,$$

in the presence of charge. Here, $\rho_{unch}$ can be found from (6a) with E=0, and the dummy variable ω has to replaced with *x* after integration in (7).

## 3. Exact Solutions

In this section, we introduce a new physical quantity Δ known as the measure of pressure anisotropy inside the stellar configuration. When we substract (6b) from (6c), we obtain the condition of anisotropic pressure

(8) $$\begin{array}{ccc} 4xZ\left(\frac{\ddot{y}}{y}\right)+2x\dot{Z}\left(\frac{\dot{y}}{y}\right)+\dot{Z}-\frac{Z-1}{x}-\frac{E^{2}}{2C} & = & \frac{\Delta }{C}, \end{array}$$

where $\Delta =p_{t}-p_{r}$ is the measure of anisotropy. If we assume that the spacetime admits a one-parameter group of conformal symmetries, then the Lie derivative $L_{X}$ along the vector field X for the metric (1) is given by

(9) $$\begin{array}{ccc} L_{X}g_{ab} & = & 2\psi g_{ab}, \end{array}$$

where $g_{ab}$ is the metric tensor field and $\psi (x^{a})$ is the conformal factor. Taking into account the static spherically symmetric spacetime (1), the vector field X can be written as

(10a)X=α(t,r)∂∂t+β(t,r)∂∂r,(10b)ψ=ψ(t,r).

The restrictions on the form of the vector X and conformal factor ψ provide a new integrability condition associated to the Weyl tensor, and this is given by the expression

(11) $$\begin{array}{ccc} L_{X}{C^{a}}_{bcd} & = & 0, \end{array}$$

where ${C^{a}}_{bcd}$ are the nonzero components of the Weyl tensor. Recently, it was showed by Manjonjo et al. [29] that the existence of a conformal symmetry (9) in the spacetime manifold, together with the integrability condition (11), lead to a relationship between the gravitational potentials *y* and *Z* known as the conformal condition

(12) $$\begin{array}{c} y=Ax^{\frac{1}{2}}\exp\left(\frac{1}{2}\sqrt{-(2n-1)}\int \frac{dx}{xZ^{\frac{1}{2}}}\right)+Bx^{\frac{1}{2}}\exp\left(-\frac{1}{2}\sqrt{-(2n-1)}\int \frac{dx}{xZ^{\frac{1}{2}}}\right), \end{array}$$

where *A*, *B* are arbitrary constants and $n<\frac{1}{2}$ is a constant related to the Weyl tensor. Note that this generates a non-conformally flat spacetime. The conformally flat spacetime corresponds to the potential

(13) $$\begin{array}{c} y=Ax^{\frac{1}{2}}\exp\left(\int \frac{dx}{xZ^{\frac{1}{2}}}\right)+Bx^{\frac{1}{2}}\exp\left(\int \frac{dx}{xZ^{\frac{1}{2}}}\right). \end{array}$$

When we substitute (12) into (8), the condition of anisotropic pressure becomes

(14) $$\dot{Z}-\frac{Z}{x}+\frac{1-n}{x}=\frac{1}{2C}\left(\Delta +E^{2}\right),$$

with $n<\frac{1}{2}$. The Einstein–Maxwell system of equations can be solved by making a specific forms for the potential *Z* and the electric field intensity *E*.

We consider the Tolman VII ansatz

(15) $$Z=1-ax+bx^{2},$$

where *a* and *b* are arbitrary constants. The choice (15) is physically reasonable as *Z* is regular at the centre (Z(0)=1) and remains positive inside the stellar body. Therefore, the potential *y* in (12) takes the form

(16) $$\begin{array}{c} y=Ax^{\frac{1}{2}}{\left(\frac{Kx}{2-ax+2\sqrt{1-ax+bx^{2}}}\right)}^{\frac{1}{2}\sqrt{-(2n-1)}}+Bx^{\frac{1}{2}}{\left(\frac{Kx}{2-ax+2\sqrt{1-ax+bx^{2}}}\right)}^{-\frac{1}{2}\sqrt{-(2n-1)}}, \end{array}$$

where *K* is a constant of integration which should satisfy the boundary conditions. A choice of the electric field intensity is required to generate a new class of exact solutions to the Einstein–Maxwell field equations. We make the following choices for the electric field function, which combined with (14) and (15), leading to expressions for the anisotropic pressure reported in Table 1.

Note from (16) that the model is singular at the center for arbitrary *n*. However, for a particular value of *n*, we can obtain a regular model. For n=0, the sphere becomes regular at the center and the gravitational potential can be written as

(17) $$\begin{array}{ccc} y & = & \frac{2B+\left(AK-aB\right)x+2B\sqrt{1-ax+bx^{2}}}{\sqrt{K}\sqrt{2-ax+2\sqrt{1-ax+bx^{2}}}}, \end{array}$$

where at the center x=0 and $y\left(0\right)=\frac{2B}{\sqrt{K}}$. We observe that with n=0 the electric field *E* and anisotropy Δ are regular, and also vanish at x=0.

Taking into account the square roots contained in the expression (17), some conditions must be imposed on the constants *a* and *b* for the regularity of the potential. Therefore, the following condition,

(18) $$\begin{array}{ccc} 2-ax+2\sqrt{1-ax+bx^{2}} & > & 0, \end{array}$$

must be satisfied. Equation (18) places a restriction on the variable *x*. We show the various conditions on *a* and *b* in terms of six cases which give the possible ranges of *x* in Table 2. We consider in particular the range in Case E where a>0 and $0<b<\frac{a^{2}}{4}$. It is possible to generate classes of exact solutions in terms of *a* and *b* for Case E.

Then, expression (14) can be solved with the help of (15) and (17) to obtain the anisotropic pressure as a function of *x* with the assumption n=0. Then, the exact solutions to the Einstein–Maxwell system of equations can be written in terms of three classes according to the choice of the electric field intensity.

### 3.1. Class I Solutions: $\frac{E^{2}}{C}=ax+dx^{2}$

When the electric field intensity corresponds to Case I reported in Table 1, we have an expression for the anisotropy function which vanishes at the center. The electric field vanishes at the centre and reaches a maximal value $\frac{E^{2}}{C}=-\frac{a^{2}}{4d}$ at the point $x=-\frac{a}{2d}$. The exact solution is given by

(19a)e2λ=11−ax+bx2,(19b)e2ν=A2K2B+(AK−aB)x+2B1−ax+bx222−ax+21−ax+bx2,(19c)ρC=3a−a2−5bx−d2x2,prC=21−ax+bx22bF1x2+F2x+F3+F3+F4x1−ax+bx2F4x2+F5x(19d)+8B+2F1x+4B1−ax+bx2−1−a+b+a2x+d2x2,(19e)pt=pr+Δ,(19f)ΔC=2b−ax−dx2,(19g)E2C=ax+dx2,(19h)σ2C=C1−ax+bx2(3a+4dx)2a+dx,

where we set

(20a)F1=AK−2aB,(20b)F2=3a2B−3aAK+4bB,(20c)F3=4(AK−aB),(20d)F4=a2B−aAK+4bB,(20e)F5=2(AK−4aB),

as new constants.

### 3.2. Class II Solutions: $\frac{E^{2}}{C}=\frac{ax}{{\left(b+ax\right)}^{2}}$

When the electric field intensity corresponds to the Case II reported in Table 1, we have another expression for the anisotropy function which also vanishes at the center. The electric field is also a vanishing quantity at the center and reaches the maximal value $\frac{E^{2}}{C}=\frac{1}{4b}$ at the point $x=\frac{b}{a}$. In this case, the exact solution is given by

(21a)e2λ=11−ax+bx2,(21b)e2ν=A2K2B+(AK−aB)x+2B1−ax+bx222−ax+21−ax+bx2,(21c)ρC=2(3a−5bx)(b+ax)2−ax2(b+ax)2,prC=21−ax+bx22bF1x2+F2x+F3+F3+F4x1−ax+bx2F4x2+F5x(21d)+8B+2F1x+4B1−ax+bx2−1+2(bx−a)(b+ax)2+ax2(b+ax)2,(21e)pt=pr+Δ,(21f)ΔC=2bxb+ax2−ax(b+ax)2,(21g)E2C=ax(b+ax)2,(21h)σ2C=aC(3b+ax)21−ax+bx2(b+ax)4,

which is a simple form.

### 3.3. Class III Solutions: $\frac{E^{2}}{C}=\frac{ax}{1+bx+dx^{2}}$

For this category of models, the electric field intensity corresponds to the Case III reported in Table 1, we obtain an expression for the anisotropy function which vanishes at the center. The electric field also vanishes at the center, and it reaches a maximal value $\frac{E^{2}}{C}=\frac{a}{2\sqrt{d}+b}$ at the point $x=\frac{1}{\sqrt{d}}$. Therefore, the exact solution has the form

(22a)e2λ=11−ax+bx2,(22b)e2ν=A2K2B+(AK−aB)x+2B1−ax+bx222−ax+21−ax+bx2,(22c)ρC=6a+6ab−10b−ax+23ad−5b2x2−10bdx321+bx+dx2−1,prC=21−ax+bx22bF1x2+F2x+F3+F3+F4x1−ax+bx2F4x2+F5x+8B+2F1x+4B1−ax+bx2−1−2a−(a−2ab+2b)x−2b2−adx2(22d)−2bdx321+bx+dx2−1,(22e)pt=pr+Δ,(22f)ΔC=2bxx(b+dx)+1−axx(b+dx)+1,(22g)E2C=ax1+bx+dx2,(22h)σ2C=aC1−(a−bx)x3+2bx+dx221+x(b+dx)3,

where $F_{1}-F_{5}$ are defined above.

The three classes of exact solutions found are given in terms of elementary functions. This helps in performing a physical analysis. All three classes of models have Δ=0 and E=0 at the stellar center which are desirable features.

## 4. Physical Quantities

In this section, we select one of the three classes of solutions obtained in this paper to generate physical quantities in terms of the coordinate *r*. The physical quantities correspond to the Class III models and the Equation (22).

### 4.1. Mass, Compactness Factor and Redshift

The exact solution (22) allows us to generate the total mass of the charged anisotropic star by inserting (22c) and (22g) into (7). Then, the following result is produced:

(23) $$M\left(r\right)=\frac{2}{3}C^{\frac{7}{4}}\left[a\left(\frac{Cr^{2}}{bCr^{2}+dC^{2}r^{4}+1}+3\right)-5bCr^{2}\right]r^{3}.$$

The compactness factor is defined by the dimensionless quantity $\mu =\frac{M}{r}$ which gives

(24) $$\mu \left(r\right)=\frac{2}{3}C^{7/4}r^{2}\left[a\left(\frac{Cr^{2}}{bCr^{2}+dC^{2}r^{4}+1}+3\right)-5bCr^{2}\right].$$

The surface redshift for a static spherically symmetric compact star is given by $Z_{s}=\frac{1}{\sqrt{1-2\mu (r)}}-1$ which takes the form

(25) $$\begin{array}{c} Z_{s}={\left[1-\frac{4}{3}aC^{7/4}r^{2}\left(\frac{Cr^{2}}{bCr^{2}+dC^{2}r^{4}+1}+3\right)+\frac{20}{4}bC^{11/4}r^{4}\right]}^{-\frac{1}{2}}-1, \end{array}$$

for the model (22).

### 4.2. Tolman–Oppenheimer–Volkoff Equation

The relativistic equation of hydrostatic equilibrium for the interior structure of the neutron star in the presence of charge and anisotropy is given by the so-called generalized Tolman–Oppenheimer–Volkoff (TOV) equation [32]:

(26) $$\begin{array}{ccc} -\left(\rho +p_{r}\right)\frac{d\nu }{dr}-\frac{dp_{r}}{dr}+\frac{2}{r}\left(p_{t}-p_{r}\right)+\frac{E}{r^{2}}\frac{d\left(r^{2}E\right)}{dr} & = & 0. \end{array}$$

This equation describes the equilibrium conditions of a charged anisotropic body under the interaction of gravitational ($F_{g}=-\left(\rho +p_{r}\right)\frac{d\nu }{dr}$), hydrostatic ($F_{h}=-\frac{dp_{r}}{dr}$), anisotropic ($F_{a}=\frac{2}{r}\left(p_{t}-p_{r}\right)$) and electrostatic ($F_{e}=\frac{E}{r^{2}}\frac{d}{dr}\left(r^{2}E\right)$) forces. They take the explicit form

(27a)Fg=−ρ+prdνdr,(27b)Fh=−dprdr,(27c)Fa=C3/2r2b1+Cr2b+Cdr2−a1+Cr2b+Cdr2,(27d)Fe=aC3/2r3+2bCr2+dC2r421+Cr2b+Cdr22.

### 4.3. Equation of State

It is possible to generate an equation of state with the help of the exact solution in the system of Equations (22a)–(22h). The Equation (22c) can be rewritten in terms of *x* as

(28) $$\begin{array}{ccc} x^{3}+\left(\frac{d\rho -3ad+5b^{2}}{5bd}\right)x^{2}+\left(\frac{2b\rho -6ab+10b+a}{10bd}\right)x+\frac{\rho -3a}{5bd} & = & 0. \end{array}$$

This is a cubic equation, but the goal is to provide a real root. The only real solution x=f(ρ) when bd≠0 is

(29) $$\begin{array}{ccc} f(\rho ) & = & {\left(30\times 2^{\frac{1}{3}}\right)}^{-1}\left[\frac{2\left(f_{2}\left(\rho \right)-4{f_{1}}^{2}\left(\rho \right)\right)}{2^{\frac{1}{3}}bd}-{\left(f_{3}\left(\rho \right)+\sqrt{{f_{3}}^{2}\left(\rho \right)+4{\left(f_{2}\left(\rho \right)-4{f_{1}}^{2}\left(\rho \right)\right)}^{3}}\right)}^{\frac{2}{3}}\right]\\ & & \times {\left[f_{3}\left(\rho \right)+\sqrt{{f_{3}}^{2}\left(\rho \right)+4{\left(f_{2}\left(\rho \right)-4{f_{1}}^{2}\left(\rho \right)\right)}^{3}}\right]}^{-\frac{1}{3}}, \end{array}$$

where

(30a)f1(ρ)=5b2−3ad+dρ,(30b)f2(ρ)=−180ab2d+30abd+300b2d+60b2dρ,(30c)f3(ρ)=−432a3d3−1080a2b2d2+540a2bd2+1800ab4d−900ab3d−10800ab2d2+2000b6−9000b4d+432a2d3+720ab2d2−180abd2−600b4d+3600b2d2ρ−144ad3+120b2d2ρ2+16d3ρ3.

Substituting (29) into (22d), we obtain the analytic expression

(31) $$\begin{array}{ccc} \frac{p_{r}}{C} & = & 2\sqrt{1-af\left(\rho \right)+bf^{2}\left(\rho \right)}\left[2bF_{1}f^{2}\left(\rho \right)+F_{2}f\left(\rho \right)+F_{3}+F_{4}f\left(\rho \right)\sqrt{1-af\left(\rho \right)+bf^{2}\left(\rho \right)}\right]\left[4f^{2}\left(\rho \right)+F_{5}f\left(\rho \right)\right.\\ & & {\left.+8B+2\left[F_{1}f\left(\rho \right)+4B\right]\sqrt{1-af\left(\rho \right)+bf^{2}\left(\rho \right)}\right]}^{-1}\\ & & -\left[2a-\left(a-2ab+2b\right)f\left(\rho \right)-2\left(b^{2}-ad\right)f^{2}\left(\rho \right)-2bdf^{3}\left(\rho \right)\right]{\left[2\left(1+bf\left(\rho \right)+df^{2}\left(\rho \right)\right)\right]}^{-1}. \end{array}$$

Therefore, we have the relationship $p_{r}=p_{r}\left(\rho \right)$ and the model satisfies a barotropic equation of state which is nonlinear in ρ.

### 4.4. Matching Conditions

The junction of the conformally symmetric interior solution to the outside solution occurs at the boundary r=R of the star. The line element for the exterior solution for a charged sphere can be written with the help of Reissner–Nordström metric

(32) $$\begin{array}{ccc} ds^{2} & = & -\left(1-\frac{2M}{r}+\frac{Q^{2}}{r^{2}}\right)dt^{2}+{\left(1-\frac{2M}{r}+\frac{Q^{2}}{r^{2}}\right)}^{-1}dr^{2}+r^{2}\left(d\theta^{2}+\sin^{2}\theta d\phi^{2}\right), \end{array}$$

where M=M(R) represents the total mass of the star and *Q* is the charge. From (1) and (32) we get

A2K2B+(AK−aB)CR2+2B1−aCR2+bC2R42(33a)×2−aCR2+21−aCR2+bC2R4−1=1−2MR+Q2R2,(33b)1−aCR2+bC2R4=1−2MR+Q2R2,

at the boundary r=R. For the matching of interior and exterior solutions, the metric potentials $e^{2\lambda }$ and $e^{2\nu }$ must be continuous at the boundary of the star where the radial pressure vanishes $p_{r}\left(r=R\right)=0$. These conditions allow us to determine the constants A and *B* and the total mass *M* as

A=KRaC94R31220bC12aR−12C2dR4+C(4(3b+1)−3C−34)R2+12CR2b+CdR2+112(34a)×2−aCR2+21−aCR2+bC2R4122B+(AK−aB)CR2+2B1−aCR2+bC2R4−1,(34b)M(R)=CR3(a−bCR2)1+bCR2+dC2R4+aCR21+bCR2+dC2R4−1,BA=K3aCR2−2bC2R4−4+aCR2−41−aCR2+bC2R4(34c)×CR23a2+4b−4abC2R4−4a+(a2+4b)CR2−4a1−aCR2+bC2R4.

The mass–radius ratio $\frac{M}{R}$ is now easily calculated. Buchdahl [40] showed that the mass–radius ratio provides important information about the physical characteristics of stellar structure.

## 5. Physical Analysis

To conserve the dimensional homogeneity and units in the physical variables, we introduce the transformation

$$r=T^{2}\tilde{r},\rho =T^{2}\tilde{\rho },p_{r}=T^{2}{\tilde{p}}_{r},p_{t}=T^{2}{\tilde{p}}_{t},$$

where the parameter T has the dimension of length. The parameters $a=\tilde{a}/T^{2}$, $b=\tilde{b}/T^{2}$ and $d=\tilde{d}/T^{2}$ are explicitly expressed in dimensions of length in ${\mathrm{km}}^{-2}$. The classes of solutions obtained in Section 4 are regular and well behaved in the interior of the star. We select the Class III solution to illustrate the physical acceptability of the model with the help of a particular choice of parameter values. Stellar masses and radii are generated for specific pulsars including: PSR J1614-2230, Vela X-1, PSR J1946+3417, 4U 1820-30, Cen X-3 and SMC X-1. For a better understanding of the nature of the matter variables throughout the stellar interior, we select PSR J1614-2230 and SMC X-1 for further study, which are the highest and the lowest in terms of numerical mass, respectively.

**Figure 1:**
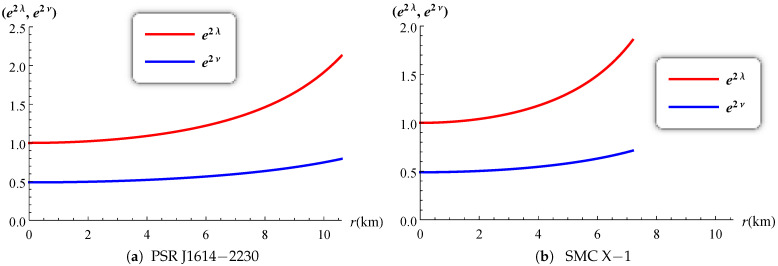
Variation of the potentials versus the radius.

**Figure 2:**
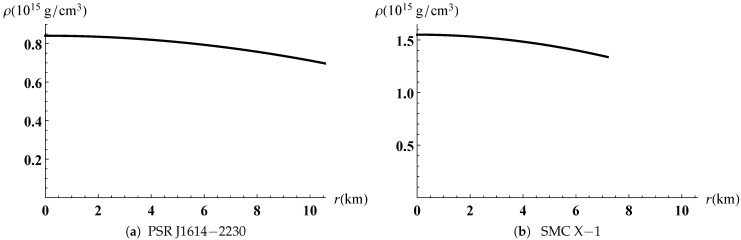
Variation of energy density versus the radius.

**Figure 3:**
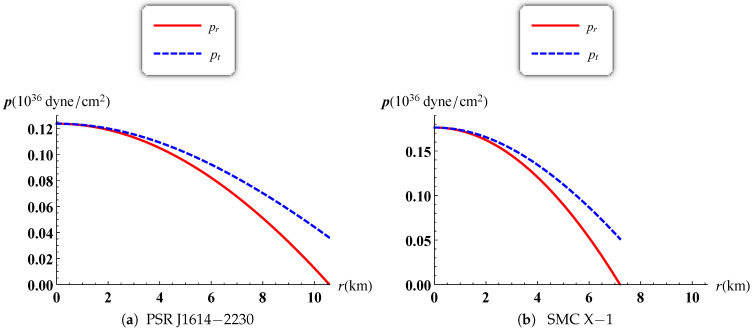
Variation of radial and tangential pressures versus the radius.

The gravitational potentials $e^{2\lambda }$ and $e^{2\nu }$ are well behaved at the center and regular throughout the stellar configuration as shown in Figure 1 for PSR J1614-2230 and SMC X-1, with the central values $e^{2\lambda (0)}=1$ and $e^{2\nu (0)}=\frac{4A^{2}B^{2}}{K}$. The energy density, the radial and tangential pressures are plotted in Figure 2 and Figure 3. The density function and the radial and tangential pressures function are positive, and monotonically decreasing with the radius. The central density $\rho \left(0\right)=\rho_{c}$ and the central pressure $p_{r}\left(0\right)=p_{t}\left(0\right)=p_{c}$ are finite and given by

$$\begin{array}{ccc} \rho_{c} & = & 3aC,\\ p_{c} & = & \frac{C\left(AK+aB\right)}{B}, \end{array}$$

**Figure 4:**
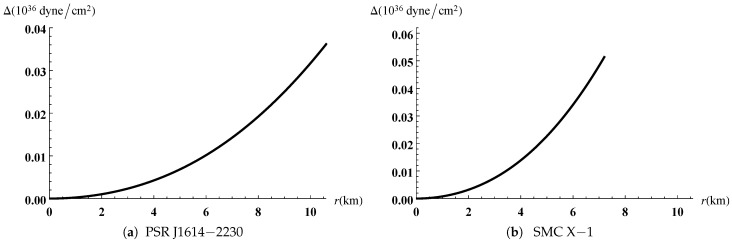
Variation of anisotropic pressures versus the radius.

**Figure 5:**
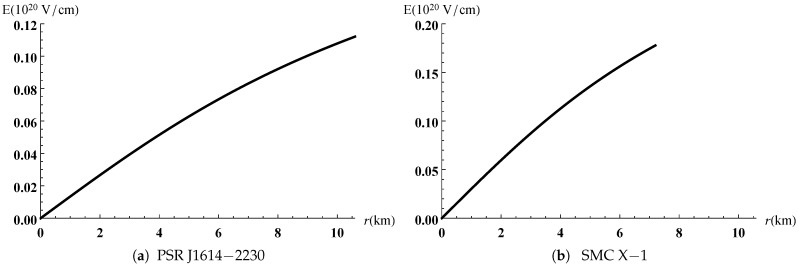
Variation of electric field versus the radius.

**Figure 6:**
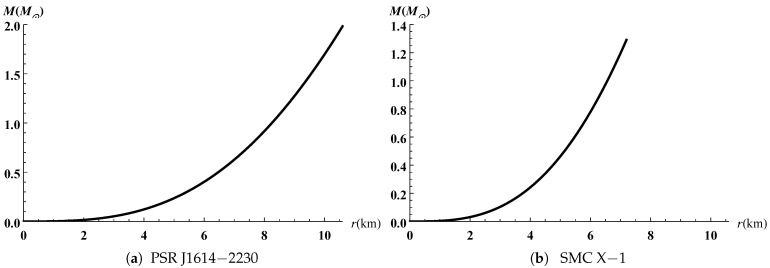
Variation of the mass versus the radius.

**Figure 7:**
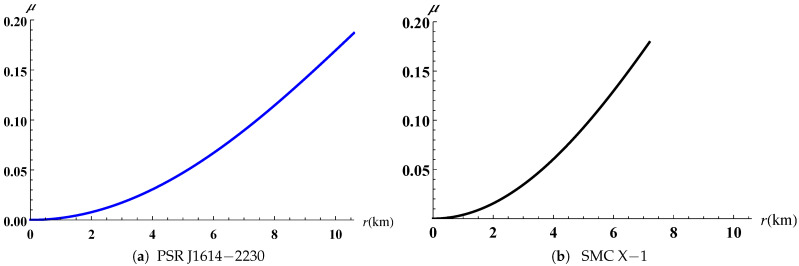
Variation of compactness factor versus the radius.

**Figure 8:**
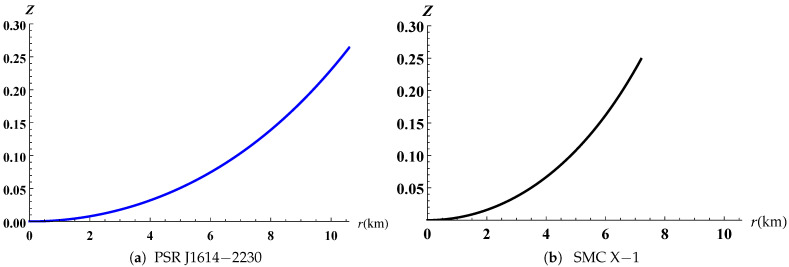
Variation of surface redshift versus the radius.

The radial pressure vanishes at the boundaries r=10.584km for PSR J1614-2230 and r=7.198km for SMC X-1. The central density $\rho_{c}=1.55\times 10^{15}\;{\mathrm{gcm}}^{-3}$ and central pressure $p_{c}=1.76\times 10^{34}\;\mathrm{dyne}{/\mathrm{cm}}^{2}$ of the compact object SMC X-1 with lower mass $M=1.04\;M_{⨀}$ are greater compared with the numerical values $\rho_{c}=8.4\times 10^{14}\;{\mathrm{gcm}}^{-3}$ and $p_{c}=1.235\times 10^{34}\;\mathrm{dyne}{/\mathrm{cm}}^{2}$ associated to PSR J1614-2230 as reported in Table 3. This behaviour has also been observed in the works of Singh et al. [35], Sharma and Ratanpal [41] and Kileba Matondo et al. [42]. The anisotropic pressure function is plotted in Figure 4 and the profiles show for both stellar candidates that it is an increasing function with increasing radius *r* and attains a maximum value at the surface. The electric field intensity presented in Figure 5 is positive and monotonically increasing for both stars selected and reaches the maximum value at the stellar surface as we observe in Table 4. The variation of mass function against the radius is reported in Figure 6 for both cases and the graph shows that it is increasing continuously throughout the star. The numerical quantities for six selected candidates presented in Table 3 are consistent with observed stars. The variation of compactness mass and redshift function are presented in Figure 7 and Figure 8 respectively, where the profiles for PSR J1614-2230 as well as SMC X-1 indicate that they are increasing with increasing radius. Referring to the Buchdahl [40] limit $\frac{2M(r)}{r}<\frac{8}{9}$, the compactness factor should satisfy the inequality $\mu =\frac{M(r)}{r}<\frac{4}{9}$. The results presented in Table 4 illustrate that $\mu =0.1865<\frac{4}{9}$ for PSR J1614-2230 and $\mu =0.1792<\frac{4}{9}$ for SMC X-1. The numerical values of the surface redshift for the objects PSR J1614-2230 and SMC X-1, as detailed in Table 4, are $Z_{s}=0.2629$ and $Z_{s}=0.2485$, respectively. These results satisfy the upper limit $Z_{s}\leq 0.9$ established by Lindblom [43] for strange stars as well as the Buchdahl [40] condition $Z_{s}\leq 2$.

**Figure 9:**
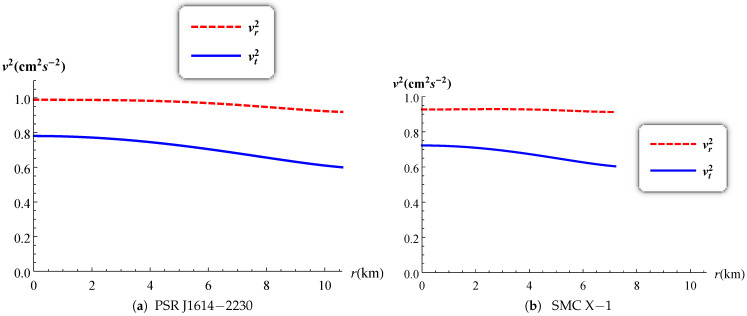
Variation of the speed of sound versus the radius.

**Figure 10:**
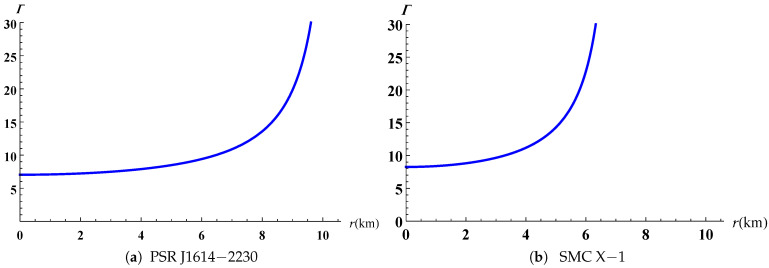
Variation of adiabatic index versus the radius.

**Figure 11:**
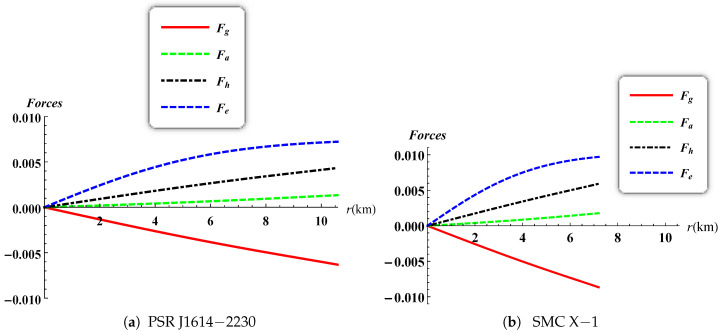
Variation of the forces versus the radius.

The radial and tangential speeds of sound have a maximum value at the stellar centre and they are decreasing monotonically throughout. The causality condition restricts them to be less than 1 ($0\leq v_{r},v_{t}\leq 1$) as reported in Figure 9 for both PSR J1614-2230 and SMC X-1. Heintzmann and Hillebrandt [44] suggested that when the pressure anisotropy is positive for a relativistic star then the stability condition satisfies the inequality Γ>4/3. The variation of the adiabatic index is plotted in Figure 10 and the profiles for both PSR J1614-2230 and SMC X-1 shows that Γ remains greater than 4/3 everywhere inside the stellar object. This allows us to confirm that our model is indeed stable. The generalized Tolman–Oppenheimer–Volkoff (TOV) equation [14,45] established above in the case of anisotropic charged fluid spheres is plotted in Figure 11. The profile shows that the static equilibrium condition is satisfied with the help of the combined effects of anisotropic, hydrostatic, electric and gravitational forces.

**Figure 12:**
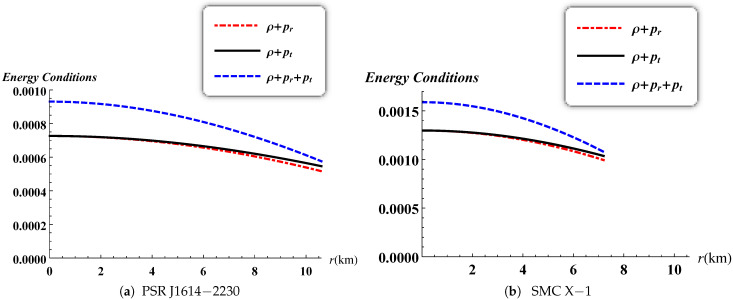
Variation of the energy conditions versus the radius.

The energy conditions play an important role in the better understanding of the nature of matter distribution inside the star. In this work, we present all the energy conditions namely: null energy condition (NEC), weak energy condition (WEC) and strong energy condition (SEC):

- NEC: $\rho +p_{i}\geq 0$,
- WEC: ρ≥0, $\rho +p_{i}\geq 0$,
- SEC: $\rho +\sum_{i}p_{i}\geq 0$,

where the index i≡[r,t] corresponds to the radial and tangential components. Figure 12 illustrates that the null energy condition, the weak energy condition and the strong energy condition are satisfied inside the stellar object for PSR J1614-2230 and SMC X-1 [46].

**Figure 13:**
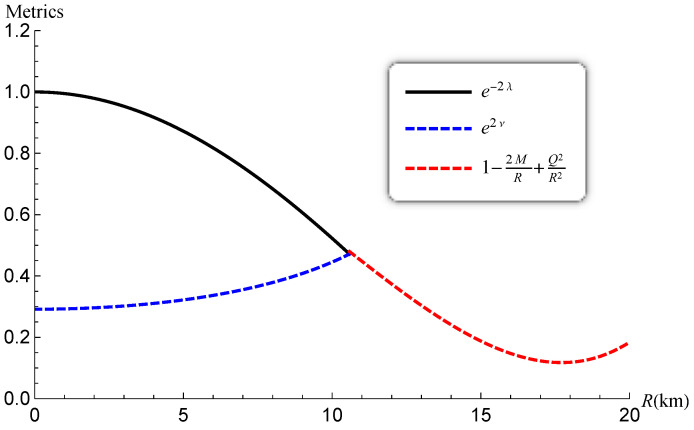
Smooth matching of the interior potentials with the Reissner–Nordström exterior for PSR J1614-2230.

The region corresponding to the interior (r<R), the exterior (r>R) and the boundary (r=R) are reported in Figure 13. The profiles indicate that $e^{2\lambda }$ and $e^{2\nu }$ are finite at the centre, regular inside the star and matches smoothly to the exterior Reissner–Nordström solution at r=10.584km.

## 6. Conclusions

In this paper, we generated three new classes of exact solutions to the Einstein–Maxwell field equations arising from the integrability of the condition of anisotropic pressure. We assumed the existence of a conformal symmetry in spacetime. This is made possible for the Tolman VII metric which has been widely studied, and shown to be well defined at the center and regular throughout the star. We have also chosen three functional forms for the electric field. We selected one of three exact solutions to perform a physical analysis. Stellar masses and radii are generated for the objects such as PSR J1614-2230, Vela X-1, PSR J1946+3417, 4U 1820-30, Cen X-3 and SMC X-1. Particular attention has been paid to the stellar objects PSR J1614-2230 and SMC X-1, which are the highest and the smallest in terms of mass, respectively, to perform a physical analysis. For particular parameter values the matter variables were plotted, and they reveal that:

- The energy density, radial and tangential pressures are monotonically decreasing functions with increasing radius, and they are positive functions inside the stellar object. The positive nature of the anisotropic pressure helps to construct a compact stellar structure.
- The causality condition is satisfied and the stability of the model is verified via the adiabatic index (Γ>4/3). The stability of the model is also verified by the equilibrium of the compact structure under the effect of anisotropic, hydrostatic, electric and gravitational forces.
- The energy conditions are satisfied everywhere inside the star.
- The masses, surface redshifts and compactness factors correspond to observed astronomical objects.

The results provided in this work reveal that our model can be used to describe a stellar interior for a charged anisotropic fluid sphere. The conformal symmetry requirement is consistent with a physically acceptable star.

## Figures and Tables

**Table 1:** Electric field and anisotropy.

Case	$\frac{E^{2}}{C}$	$\frac{\Delta }{C}$
I	$ax+dx^{2}$	$2bx-\frac{2n}{x}-\left(ax+dx^{2}\right)$
II	$\frac{ax}{{\left(b+ax\right)}^{2}}$	$2bx-\frac{2n}{x}-\frac{ax}{{\left(b+ax\right)}^{2}}$
III	$\frac{ax}{1+bx+dx^{2}}$	$2bx-\frac{2n}{x}-\frac{ax}{1+bx+dx^{2}}$

**Table 2:** Conditions on *a* and *b* for the range of *x*.

Case	Conditions and Range
A	a<0, b<0
	$-\frac{1}{2b}\left(\sqrt{a^{2}-4b}-a\right)\leq x\leq \frac{1}{2b}\left(\sqrt{a^{2}-4b}+a\right)$
B	a<0, $0<b<\frac{a^{2}}{4}$
	$x\geq \frac{1}{2b}\left(\sqrt{a^{2}-4b}+a\right)$
C	a<0, $b=\frac{a^{2}}{4}$
	$x>-\frac{1}{2b}\left(\sqrt{a^{2}-4b}-a\right)$
D	a>0, b<0
	$-\frac{1}{2b}\left(\sqrt{a^{2}-4b}-a\right)\leq x\leq \frac{1}{2b}\left(\sqrt{a^{2}-4b}+a\right)$
E	a>0, $0<b<\frac{a^{2}}{4}$
	$x\leq -\frac{1}{2b}\left(\sqrt{a^{2}-4b}-a\right)$
F	a>0, $b=\frac{a^{2}}{4}$
	$x<-\frac{1}{2b}\left(\sqrt{a^{2}-4b}-a\right)$

**Table 3:** Masses for d=−8, A=15.10, B=3.5, A=0.2, and K=4.

*a*	*b*	*C*	R	r (km)	$M\;(M_{⨀})$	Star
$2\sqrt{12}$	8.00	0.179	15.40	10.584	1.974	PSR J1614-2230
$2\sqrt{12.5}$	7.90	0.184	15.20	9.823	1.77	Vela X-1
$2\sqrt{12.8}$	8.00	0.186	15.00	9.400	1.657	PSR J1946+3417
$2\sqrt{13.1}$	8.50	0.200	14.65	8.716	1.585	4U 1820-30
$2\sqrt{13.12}$	8.13	0.199	14.13	8.329	1.49	Cen X-3
$2\sqrt{13.15}$	8.18	0.200	12.26	7.198	1.29	SMC X-1

**Table 4:** Values of the physical quantities at the center and surface.

Star	$\rho_{c}\;({\mathrm{gcm}}^{-3})$	$\rho_{s}\;({\mathrm{gcm}}^{-3})$	$p_{c}\;(\mathrm{dyne}{/\mathrm{cm}}^{2})$	E (V/cm)	μ	$Z_{s}$
PSR J1614-2230	$0.84\times 10^{15}$	$0.6964\times 10^{15}$	$0.1235\times 10^{35}$	$0.22\times 10^{20}$	0.1865	0.2629
PSR J1946+3417	$0.95\times 10^{15}$	$0.8138\times 10^{15}$	$0.1170\times 10^{35}$	$0.21\times 10^{20}$	0.1763	0.2429
SMC X-1	$1.55\times 10^{15}$	$1.3380\times 10^{15}$	$0.1760\times 10^{35}$	$0.17\times 10^{20}$	0.1792	0.2485

## Data Availability

This manuscript has no associated data.
